# Supplementary material for: Symmetry breaking in optimal transport networks
Source: Nat Commun. 2024 May 4;15:3758. doi: 10.1038/s41467-024-48068-9 (PMC11069546; doi:10.1038/s41467-024-48068-9)
Supplement: Supplementary file 1 — Supplementary Information [file 41467_2024_48068_MOESM1_ESM.pdf]

# Supplementary Information for "Symmetry breaking in optimal transport networks"

## I. MULTIPLEX TRANSPORTATION MODEL

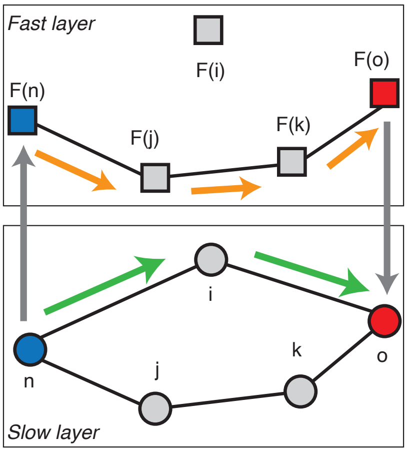


FIG. S1. **Illustration of the multiplex transportation model.** In the slow layer, the cost of an edge equals one; in the fast layer, the cost of an edge is reduced by the factor $0 \leq \eta \leq 1$. Replica nodes across layers are connected by edges with cost $c \geq 0$. Two possible paths connecting node $n$ (blue circle) to node $o$ (red circle) are highlighted. The path $n \to i \to o$ has cost equal to 2 as it uses only two edges in the slow layer (green arrows). The second path, i.e., $n \to F(n) \to F(j) \to F(k) \to F(o) \to o$, involves two changes of layer (gray arrows), and a path in the fast layer (orange arrows). Its total cost is $3\eta + 2c$, as each of the three edges used in the fast layer has cost equal to $\eta$, and $2c$ is the cost required to switch layer twice. The second path is more convenient than the first one as long as $3\eta + 2c < 2$.

We consider a multiplex network composed of a slow layer and a fast layer. For a schematic example of such a system, see Fig. S1; for a list of variables/metrics used to characterize the model, see Table S1. We denote with $\mathcal{G}$ the set of nodes in the slow layer, and with $\mathcal{H}$ the set of nodes in the fast layer. Both layers contain $N$ nodes, i.e., $|\mathcal{G}| = |\mathcal{H}| = N$. Each node in the slow layer has a one-to-one correspondence with a node in the fast layer; we indicate with $F(\cdot)$ the map between labels of nodes across layers, i.e., if $n \in \mathcal{G}$ then $F(n) \in \mathcal{H}$, and vice versa. We denote with $\mathcal{S}$ and $\mathcal{F}$ the set of edges of the slow and the fast layer, respectively. Each edge in the fast layer has a replica edge in the slow layer, i.e., $(F(n), F(m)) \in \mathcal{F}$ implies $(n, m) \in \mathcal{S}$, but the vice versa is not necessarily true. The weight of each edge in the slow layer equals one, i.e., $w_{n,m} = 1$ for all $(n, m) \in \mathcal{S}$, whereas the weight associated with the edges of the fast layer is equal to $0 \leq \eta \leq 1$, i.e., $w_{F(n),F(m)} = \eta$ for all $(F(n), F(m)) \in \mathcal{F}$. Replica nodes are connected to each other by edges of weight $c \geq 0$, i.e., $w_{n,F(n)} = c$ for all $n \in \mathcal{G}$.

When considered in isolation, the slow layer form a single connected component, whereas the fast layer is not necessarily connected. The connectedness of the slow layer implies, however, that in the overall system, composed of the interconnected slow and fast layers, there exists at least a path connecting any pair of nodes. The cost of a path, e.g., $n \to F(n) \to \ldots \to F(k) \to k \to \ldots \to m$, between two nodes $n$ and $m$ in the network is given by the sum of the weights of all edges that compose the path. We denote with $d_{n\to m}$ the cost of the minimum-cost path between nodes $n$ and $m$. Naturally, the same definition of cost applies to paths between any pairs of nodes, belonging to either the slow or the fast layer. For example, $d_{F(n)\to m}$ denotes the cost of the minimum-cost path between $F(n) \in \mathcal{H}$ and $m \in \mathcal{G}$. The minimum-cost path between two nodes can either use edges in the slow layer only, or take advantage of some of the edges in the fast layer (see Figure S1). In particular, the path $n \to F(n) \to F(m) \to \ldots \to F(r) \to F(s) \to s$ composed of $\ell$ edges in the fast layer only is preferred to its replica path $n \to m \to \ldots \to r \to s$ whenever $\ell$ is larger than the critical value

$$r_c = \frac{2c}{1-\eta} \; . \qquad \text{(S1)}$$

## II. OPTIMIZATION OF THE FAST LAYER

We identify a special node in the slow layer of the network, i.e., the center of the network. The label of the center is $o$. We denote with $d_n := d_{n\to o}$ the cost of the minimum-cost path of the generic node $n$ to $o$. Also, we assume that each node $n$ in the slow layer has associated a weight $p_n \geq 0$, representing the demand of node $n$. We then define the weighted average cost to the center as

$$\tau(\mathcal{F}) = \frac{\sum_{n\in\mathcal{G}} d_n \, p_n}{\sum_{n\in\mathcal{G}} p_n} \; . \qquad \text{(S2)}$$

We stress that the above function is computed over all nodes in the slow layer only, but eventual minimum-cost paths can take advantage of edges in the fast layer. Clearly, $\tau$ depends on the various parameters of the model. In Eq. (S2), we explicit, on purpose, only the dependence of $\tau$ on the fast layer $\mathcal{F}$ as this is the primary object of our investigation. We consider in fact the optimization problem aimed at finding the best set of edges in the fast layer able to minimize the objective function of Eq. (S2). The minimization is constrained by the number of edges $L$ that are in the fast layer, where $L$ is measured in the same units of cost as $\tau$ and $c$. Specifically, we aim at solving

$$\mathcal{F}^* = \arg \min_{|\mathcal{F}|=L} \tau(\mathcal{F}) \; , \qquad \text{(S3)}$$

| notation | object represented |
|---|---|
| $\mathcal{G}$ | set of nodes in the slow layer |
| $\mathcal{S}$ | set of edges in the slow layer |
| $\mathcal{H}$ | set of nodes in the fast layer |
| $\mathcal{F}$ | set of edges in the fast layer |
| $N = \|\mathcal{G}\| = \|\mathcal{H}\|$ | size of the network |
| for all $n \in \mathcal{G} \Leftrightarrow F(n) \in \mathcal{H}$ | one-to-one map of nodes across layers |
| $w_{n,m} = 1$ for all $(n, m) \in \mathcal{S}$ | weight of the edges in the slow layer |
| $w_{F(n),F(m)} = \eta$ for all $(F(n), F(m)) \in \mathcal{F}$ | weight of the edges in the fast layer |
| $w_{n,F(n)} = c$ for all $n \in \mathcal{G}$ | weight of interlayer edges or switching cost |
| $r_c = 2c/(1-\eta)$ | critical cost of the model, see Eq. (S1) |
| $d_{n\to m}$ for all $n, m \in \mathcal{G} \cup \mathcal{H}$ | cost of the minimum-cost from node $n$ to node $m$ |
| $o \in \mathcal{G}$ | center of the network |
| $d_n := d_{n\to o}$ for all $n \in \mathcal{G} \cup \mathcal{H}$ | cost of the minimum-cost path of node $n$ to $o$ |
| $p_n$ for all $n \in \mathcal{G}$ | weight of nodes in the slow layer |
| $\tau(\mathcal{F})$ | objective function, see Eq. (S2) |
| $s_{n,m}(\mathcal{F})$ for all $(n, m) \in \mathcal{S}$ and $(F(n), F(m)) \notin \mathcal{F}$ | marginal gain in the objective function, see Eq. (S4) |
| $\mathcal{F}^*$ | optimal configuration of the fast layer, see Eq. (S3) |
| $k^*$ | number of main branches of the optimal layer, see Eq. (??) |

TABLE S1. List of variables and metrics used in the description of the multiplex transportation model and its associated optimization problem. For each of them, we report the notation used thorough the document and the quantity that is meant to represent.

where we indicated with $|\mathcal{F}|$ the number of edges in $\mathcal{F}$, and once more we did not write the explicit dependence of the objective function from the structure of the slow layer $\mathcal{S}$, the center of the network $o$, the weights $p_n$ for all nodes $n \in \mathcal{G}$, and the values of the parameters $\eta$ and $c$.

Finding the exact solution to the optimization problem of Eq. (S3) is computationally infeasible as it requires to test all possible $\binom{|\mathcal{S}|}{L}$ configurations of the fast layer. However, the number of suitable configurations for $\mathcal{F}^*$ can be restricted by some properties that the optimal layer must satisfy.

## III. PROPERTIES OF THE OPTIMAL FAST LAYER

We show that the optimal fast layer $\mathcal{F}^*$, solution of the optimization problem of Eq. (S3), is a tree containing at least one edge incident to node $F(o)$, i.e., the replica node of the center of the network.

### Connectedness

Assume that the edges in the fast layer $\mathcal{F}$ generate a graph consisting of at least one component that has no edges incident to node $F(o)$. Focus on one of these components, and indicate the set of its edges as $\mathcal{C} \subseteq \mathcal{F}$.

Consider now the edge $(F(m), F(n)) = \arg\max_{(F(i),F(j))\in\mathcal{C}} \max\{d_{F(i)}, d_{F(j)}\}$, i.e., the edge in the component that corresponds to the largest value of the cost of the minimum-cost path to the center $o$. Without loss of generality, suppose that $d_{F(m)} \geq d_{F(n)}$. Based on our premise, the minimum-cost path from node $F(m)$ to $o$ can be written as $F(m) \to F(n) \to \cdots \to F(s) \to F(r) \to r \to q \to \cdots \to o$, meaning there is at least one edge $(r, q) \in \mathcal{S}$ in the minimum-cost path to $o$ such that $(F(r), F(q)) \notin \mathcal{F}$.

Define the set $\mathcal{F}' = \mathcal{F} \setminus (F(m), F(n)) \cup (F(r), F(q))$, i.e., the same set as $\mathcal{F}$ but with the edge $(F(m), F(n))$ replaced by $(F(r), F(q))$. We have $\tau(\mathcal{F}') \leq \tau(\mathcal{F})$. In fact, the cost of the minimum-cost path to $o$ of every node $n \in \mathcal{S}$ whose minimum-cost path to $o$ utilizes the edge $(F(m), F(n))$ when the fast layer is $\mathcal{F}$ will no increase when the fast layer is $\mathcal{F}'$; however, some of the other nodes that do not use the edge $(F(m), F(n))$ to reach $o$ when the fast layer is $\mathcal{F}$ can decrease the cost of their minimum-cost path by using the edge $(F(r), F(q))$ when the fast layer is $\mathcal{F}'$.

We note that, when passing from $\mathcal{F}$ to $\mathcal{F}'$, the deletion of the edge $(F(m), F(n))$ does lead to any split of the component $\mathcal{C}$, except for the potential removal of node $F(m)$; however, the addition of the edge $(F(r), F(q))$ can lead to the merger of $\mathcal{C}$ with another component, as well as to the inclusion of an edge incident to $F(o)$.

In summary, if the fast layer $\mathcal{F}$ is formed by at least one component that does not contain any edge incident to $F(o)$, then we can always find another configuration of the fast layer that is better than $\mathcal{F}$. The procedure can be iterated until its premise is no longer true. As a result, the optimal fast layer $\mathcal{F}^*$ must contain only one component with at least one edge incident to $F(o)$.

### Tree structure

Suppose the fast layer $\mathcal{F}$ contains a loop. If the loop is formed by an odd number of edges, then there is an edge $(F(n), F(m))$ for which $d_{F(n)} = d_{F(m)}$. This edge is irrelevant for $\tau(\mathcal{F})$, as no minimum-cost path passes through it. In fact, suppose node $i$ is such that $d_{i \to F(n)} \leq d_{i \to F(m)}$. Then, we can write $d_i = d_{i \to F(n)} + d_{F(n)} \leq d_{i \to F(m)} + d_{F(m) \to F(n)} + d_{F(n)}$. Similarly, if the loop has an even number of edges then there exists a node $F(n)$ such that there are two minimum-cost paths from the node $F(n)$ to $o$. One of the edges incident to $F(n)$ in the loop can be removed without affecting $\tau(\mathcal{F})$. In both cases, the removed edge can be replaced by another edge potentially able to decrease $\tau$, thus the optimal fast layer $\mathcal{F}^*$ should not contain any loops.

## IV. OPTIMIZATION TECHNIQUES

In the previous sections, we proved that the optimization problem of Eq. (S3) can be solved by looking only at fast-layer configurations consisting of trees that contain at least one edge incident to the replica of the center of the network. This fact dramatically reduces the number of potential configurations that one should look at, however, it does not address the computational unfeasibility of the optimization problem. In this section, we introduce numerical techniques able to approximate solutions to the problem in an efficient and effective manner.

### A. Greedy optimization

The algorithm takes as inputs the slow layer $\mathcal{S}$, the parameters $c$ and $\eta$, and the desired size $L$ of the fast layer. The output is the fast layer $\mathcal{F}_g$, representing a greedy solution to the optimization problem of Eq. (S3).

We initially set $\mathcal{F}_g = \emptyset$, and we compute the cost of the minimum-cost path of all nodes to the center $o$. This information is stored in the vector $\vec{d}$. Also, we initialize $s_{n,m}(\mathcal{F}_g)$ for all edges $(n, m) \in \mathcal{S}$ such that $(F(n), F(m)) \notin \mathcal{F}_g$. $s_{n,m}(\mathcal{F}_g)$ quantifies the change in the objective function of Eq. (S2) that would be induced by adding the edge $(F(n), F(m)$ to $\mathcal{F}_g$, i.e.,

$$s_{n,m}(\mathcal{F}_g) = \tau(\mathcal{F}_g) - \tau(\mathcal{F}_g \cup (F(n), F(m))) \ . \quad \text{(S4)}$$

Then, we iterate the following:

1. We solve

$$(i, j) = \arg \max_{(n,m) \in \mathcal{S} | (F(n), F(m)) \notin \mathcal{F}_g} s_{n,m}(\mathcal{F}_g) \ , \quad \text{(S5)}$$

   i.e., we find the edge corresponding to the largest drop in the objective function. Eventual ties are randomly broken.

2. We update $\mathcal{F}_g \to \mathcal{F}_g \cup (F(i), F(j))$. Also, we update the entries of the vector $\vec{d}$ using a suitably modified Dijkstra's algorithm.

3. If $|\mathcal{F}_g| = L$, we exit from the algorithm.

4. We update the scores $s_{n,m}(\mathcal{F}_g)$ for all $(n, m) \in \mathcal{S}$ such that $(F(n), F(m)) \notin \mathcal{F}_g$, and we go back to point 1. Please note that the update of each score $s_{n,m}(\mathcal{F}_g)$ also relies on the suitably modified Dijkstra's algorithm of point 2.

The algorithm outputs not just the greedy solution $\mathcal{F}_g$, but also the cost of the minimum-cost path of all nodes to the center $\vec{d}$ as well as the value of the objective function $\tau(\mathcal{F}_g)$.

#### *Speeding up the greedy algorithm*

In the naive implementation described above, a significant number of computations are performed to update the scores $s_{n,m}(\mathcal{F}_g)$ for all edges in the slow layer. However, many of these updates are not required, and the algorithm can be speeded up quite significantly.

First, we know that the optimal fast layer is a tree containing at least one edge incident to $F(o)$, thus not all edges of the slow layer $\mathcal{S}$ should be considered at each stage of the algorithm. At the first iteration, only replica of edges that are incident to $F(o)$ should be considered; then in the following iterations, only replica edges that are incident to previously added edges or node $F(o)$ and that do not close eventual loops in the fast layer should be considered.

Second and more important, there is no need to update the score of all potential edges that can be added. This follows from the fact that the score $s_{n,m}(\mathcal{F}_g)$ associated to the edge $(F(n), F(m)) \notin \mathcal{F}_g$, that is incident to another edge that has been already added to $\mathcal{F}_g$, does not increase as the number of iterations of the greedy algorithm increases. We therefore rely on a lazy-search procedure [**?** ]. Specifically, we keep a sorted list (i.e., heap data structure) of the scores of all edges that are incident to edges in $\mathcal{F}_g$. When the new edge $(F(n), F(m))$ enters in the list, we compute its score $s_{n,m}(\mathcal{F}_g)$ and insert it in a temporary buffer. We then set $s_{\max} = s_{n,m}(\mathcal{F}_g)$. We pop out one edge at a time from the sorted list in descending order. Given an edge $(r, s)$, we update its score only if the current score $s_{r,q}(\mathcal{F}_g) > s_{\max}$. If updated, we insert the new score in the temporary buffer. If the updated score is $s_{r,q}(\mathcal{F}_g) \geq s_{\max}$, then we set $s_{\max} = s_{r,q}(\mathcal{F}_g)$ and go to the next element in the sorted list. Otherwise, we stop with the update, as we already found the edge with the largest score, i.e, the solution of Eq. (S5). We finally put back elements from the temporary buffer to the sorted list.

The only exception to the above rule of a score decreasing as the number of iterations of the algorithm increases is for an edge that can potentially close a loop. However,

we know that the optimal fast layer should not contain loops, thus edges of this type are not considered in our search.

If the score of $K$ edges $\{(n_1, m_1), \ldots, (n_K, m_K)\}$ is updated, we first compute their scores and insert them in the temporary buffer. Then, we find the one with the maximum score, say $(n, m)$. We simply set $s_{\max} = s_{n,m}(\mathcal{F}_g)$, and repeat the same instructions as above to perform our lazy search.

### *Modified Dijkstra's algorithm*

At step 2 of the greedy optimization algorithm, we mentioned that the components of the vector of the minimum-cost values $\vec{d}$ are updated using a modified Dijkstra's algorithm. This procedure is used also to estimate potential changes to the vector $\vec{d}$, thus in the estimate of the scores $s_{n,m}(\mathcal{F}_g)$ at step 4 of the greedy optimization. Our modified Dijkstra's algorithm is designed to perform local updates, as not all components of the vector $\vec{d}$ will necessarily change after the new edge $(F(i), F(j))$ is added to $\mathcal{F}_g$. Without loss of generality, let's assume that before the addition of the edge, node $F(i)$ has degree equal zero in the fast layer, whereas the degree of node $F(j)$ is larger than zero. We first update $d_{F(i)} \to \min\{d_{F(i)}, d_i + c, \min_{(i,q)\in\mathcal{F}_g} d_{F(q)} + \eta\}$. The three elements corresponds respectively to: (i) unchanged cost of the minimum-cost path, (ii) cost of the minimum-cost path reduced by taking the slow layer, (iii) cost of the minimum-cost path reduced by taking the fast layer. We then update the components of the other nodes using a Dijkstra-like algorithm. The algorithm is started from node $F(i)$, and exploits edges in both $\mathcal{S}$ and $\mathcal{F}_g$ leading therefore to updates in the vector $\vec{d}$ that regards nodes in both the slow and the fast layer. However, it considers only moves $q \to p$ such that $d_q + w_{q,p} \leq d_p$; in such a case, the component of the node $p$ is updated as $d_p \to d_q + w_{q,p}$. The Dijkstra-like algorithm does not necessarily visit all nodes in the network, but only those whose component in the vector $\vec{d}$ is decreased by the addition of the edge $(F(i), F(j))$.

### *Submodularity of the objective function*

If the fast layer is composed of a single connected component, the score $s_{n,m}(\mathcal{F}_g)$ associated with each potential edge $(F(n), F(m))$ that could be added to the set $\mathcal{F}_g$ is a non-increasing function of the number of edges already added to $\mathcal{F}_g$. This fact follows from the simple observation that the only effect that the addition of the edge $(F(n), F(m))$ can have is adding novel minimum-cost paths in the network, potentially reducing the cost of the minimum-cost path of some nodes. The drop in the cost of the minimum-cost path induced by the addition of the edge $(F(n), F(m))$ is maximal when $\mathcal{F}_g = \emptyset$. If $\mathcal{F}_g \neq \emptyset$, however, the drop is potentially reduced given that the other edges already in the set can provide minimum-cost paths to the center that do not pass through the edge $(F(n), F(m))$.

As a matter of fact, we can write

$$s_{n,m}(\mathcal{F}') \begin{array}{l} = \tau(\mathcal{F}') - \tau(\mathcal{F}' \cup (F(n), F(m))) \\ \geq \tau(\mathcal{F}'') - \tau(\mathcal{F}'' \cup (F(n), F(m))) \\ = s_{n,m}(\mathcal{F}'') \end{array} , \qquad \text{(S6)}$$

where $\mathcal{F}' \subseteq \mathcal{F}''$, both $\mathcal{F}'$ and $\mathcal{F}''$ are trees with at least one edge incident to $F(o)$, and $(F(n), F(m))$ is an arbitrary edge that is incident to one edge in both $\mathcal{F}'$ and $\mathcal{F}''$.

In summary, the function $\tau$ that is optimized is a non-negative, non-increasing, submodular function. As such, the proposed greedy algorithm allows us to find solutions that are at maximum a factor $(1 - 1/e) \simeq 0.63$ above the ground-truth optimum [**?** ].

The only exception to the inequality (S6) is when both $F(n)$ and $F(m)$ are adjacent to edges in $\mathcal{F}''$. This is, however, not possible as the edge $(F(n), F(m))$ would close a loop, which is in contradiction with the fact that the optimal fast layer must be a tree.

### *Selecting greedy solutions*

In the above formulation of the greedy optimization algorithm, we tacitly assumed that $r_c < 1$, meaning that the addition of every edge to the fast layer can potentially reduce the value of the objective function. This fact allows us to initialize the algorithm with $\mathcal{F}_g = \emptyset$.

A simple way to re-use the previous algorithm for $r_c \geq 1$ is changing the initialization. Specifically, we can proceed by first identifying a node $n \in \mathcal{G}$ for which $d_n = \lfloor r_c \rfloor$ and one of the minimum-cost paths connecting $n$ to $o$, and then adding $(F(i), F(j))$ to $\mathcal{F}_g$ for each edge $(i, j) \in \mathcal{S}$ that is part of such a minimum-cost path.

We adopt a different protocol that can be used for any value of $r_c$. Although the switching cost $c$ is an input of the optimization problem, we treat it as a variable by considering $M = 1,000$ different values in the interval $[0, (1 - \eta)/2]$. For each of them, we find a solution using the greedy optimization algorithm, namely $\mathcal{F}_g^{(1)}, \ldots, \mathcal{F}_g^{(M)}$. We compute the value of the objective function associated to each of these sets by using the input value of the switching cost $c$, and find the best solution by identifying the one corresponding to the smallest value of the objective function.

### *Complexity of the greedy algorithm*

We show the computational complexity of the greedy algorithm after implementing the speed-ups described above. We use the two-dimensional multiplex transportation model introduced in the main manuscript with

the radius of the triangular lattice equal to $R$. We generate greedy solutions for $c = 0.1$, $\eta = 0.1$, and $L = R$. The time required to obtain the greedy solution is plotted against the radius $R$ in Figure S2. Note that the multiplex transportation model with radius $R$ has $N = 3R^2 + 3R + 1$ nodes in each layer.

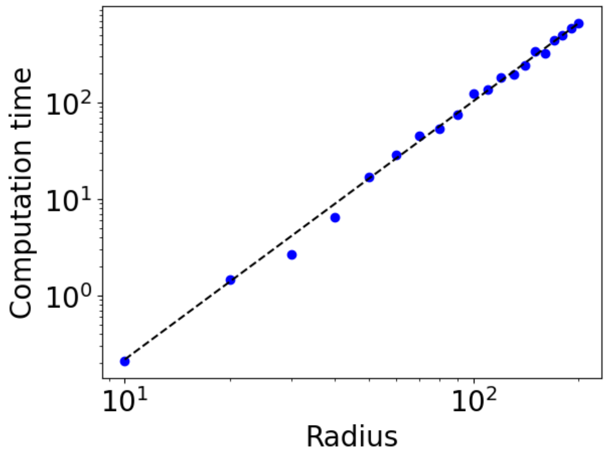


FIG. S2. **Computational complexity of the greedy algorithm.** We generate slow layers according to our multiplex transportation model with variable triangular lattice radius $R$. We set the parameters of the model as $L = R$, $\eta = 0.1$, and $c = 0.1$. We then apply the greedy algorithm to approximates solutions to the optimization problem of Eq. (S3). We plot the computational time $V$ required by the greedy algorithm as a function of $R$. Time is measured in seconds. Simulations were run on an Intel(R) Xeon(R) CPU E5-2690 v4 @ 2.60 GHz. The dashed line stands for $V \sim R^{2.7}$.

### B. Simulated Annealing

We use a simulated-annealing scheme to approximate solutions to the optimization problem of Eq. (S3). Specifically, we start from a set $\mathcal{F}_{\rm sa}$ constructed by sequentially adding $L$ randomly chosen edges to the fast layer such that no loops are present, the graph is connected and at least one edge is incident to node $F(o)$. We estimate the objective function $\tau(\mathcal{F}_{\rm sa})$. We impose the cooling factor $\gamma = 0.999$, the minimum temperature $T_{\rm min} = 10^{-3}$, and the initial temperature $T = 10^2$. We then iterate:

1. Select a random edge $(F(n), F(m)) \in \mathcal{F}_{\rm sa}$ such that either the degree of node $F(n)$ or $F(m)$ equals one in the fast layer. Select a random edge $(i, j) \in \mathcal{S}$ such that the sum of the degrees of nodes $F(i)$ and $F(j)$ in the fast layer is equal to one.

2. Consider the set $\mathcal{F}'_{\rm sa} = \mathcal{F}_{\rm sa} \setminus (F(n), F(m)) \cup (F(i), F(j))$. Please note that $\mathcal{F}'_{\rm sa}$ is still a compatible solution of the optimization problem being a connected tree composed of $L$ edges with at least one edge incident to node $F(o)$. Compute $\tau(\mathcal{F}'_{\rm sa})$.

3. With probability $\min\{1, e^{[\tau(\mathcal{F}'_{\rm sa}) - \tau(\mathcal{F}_{\rm sa})]/T}\}$, we accept the change and update $\mathcal{F}_{\rm sa} \to \mathcal{F}'_{\rm sa}$.

4. Update the temperature as $T \to \gamma T$. If $T > T_{\rm min}$, go back to point 1, otherwise end the algorithm.

## V. RESULTS

### A. Erdős-Rényi graphs

In Figure S3, we consider a slow layer formed by the edges of a single instance of the Erdős-Rényi (ER) model with $N = 1,000$ and average degree $\langle k \rangle = 4$. As the center of the network, we use the node with the largest degree equal to 11 in this specific realization of the ER model. In the objective function of Eq. (S2), we assume a weight $p_n$ =const. for all nodes $n \in \mathcal{G}$. We use the greedy optimization algorithm to approximate solutions to the optimization problem of Eq. (S3) for $L = 10$ and various combinations of the model parameters $c$ and $\eta$. The heat map displays the corresponding values of $k^*$ as a function of the model parameters. No optimal fast layer can be constructed if $L \leq r_c$. For $L > r_c$, we observe instead a rich phase diagram where $k^*$ ranges between 1 and 5. In general, for a fixed value of $\eta$ and sufficiently large $c$, we always observe $k^* = 1$.

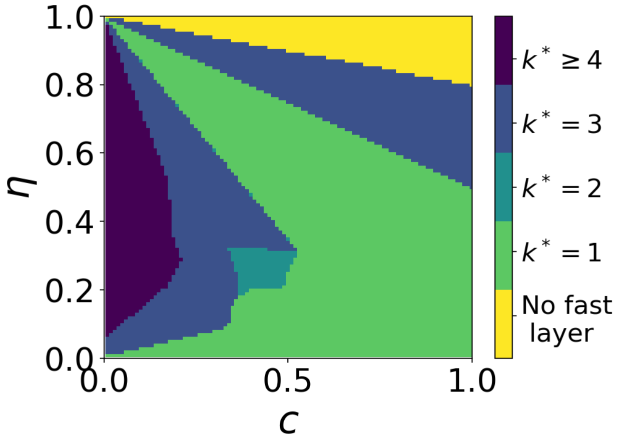


FIG. S3. **Phase diagram for random networks.** We show the heat map illustrating the type of optimal solutions obtained for different combinations of $c$ and $\eta$. We consider a slow layer consisting of an ER network of size $N = 1000$ and set the size of the fast layer solution to $L = 10$. We plot the number of branches $k^*$ observed in the optimal fast layer as a function of the model parameters $\eta$ and $c$. The yellow region indicates $L \leq r_c$. In this regime, there is no benefit in building a fast layer.

### B. Two-dimensional lattices

In Figure S4, we report results concerning the optimal fast-layer configuration obtained on two-dimensional lat-

tices when the weight $p_n$ associated to node $n \in \mathcal{G}$ is

$$p_n \sim e^{-d(n,o)} \ , \quad \text{(S7)}$$

where $d(i,o)$ is the Euclidean distance between nodes $n$ and $o$. We generate phase diagrams similar to Figures 2 e and f in the main paper but for weights obeying Eq. (S7), see Figure S5.

## VI. CONTINUOUS-SPACE APPROXIMATIONS

### A. One-dimensional system

We consider a slow layer whose center is located in the origin, and formed of two segments of length $R$ extending symmetrically to the left and the right of the center (see Figure 1 in the main text). We model the fast layer as (i) a connected segment that is (ii) attached to the replica of the origin of the lattice. Properties (i) and (ii) are satisfied by the optimal layer also in continuous space. We omit the proof as this is very similar to the one valid in discrete space. The fast layer extends to the right of the origin with a segment of length $\alpha L$ and to the left with a segment of length $(1-\alpha)L$, where $0 \leq \alpha \leq 1/2$ is a tunable parameter and $L \leq R$ is the total length of the fast layer. The goal of our calculation below is to find the value $\alpha^*$ corresponding to the optimal configuration of the fast layer, i.e., the solution of the continuous-space approximation of the optimization problem of Eq. (S3).

#### Main calculation

In the following, we indicate with $p(x)$ the continuous version of $p_n$. We will assume that $p(x)$ is a non-negative and even function, i.e., $p(x) \geq 0$ and $p(x) = p(-x)$ for all $x \in [-R, R]$. Further, we make use of the formula of integration by parts

$$\int dx \, x \, p(x) = x \, G(x) - H(x) \ , \quad \text{(S8)}$$

where

$$G(x) = \int dx \, p(x) \quad \text{(S9)}$$

is the indefinite integral of the function $p(x)$, and

$$H(x) = \int dx \, G(x) \quad \text{(S10)}$$

is the indefinite integral of the function $G(x)$. We note that in our problem $p(x) \geq 0$ for all $x$, thus $G(x)$ and $H(x)$ are non-decreasing functions.

As we mentioned, the fast layer is composed of a segment of length $\alpha L$ extending to the right of the center, and $(1-\alpha)L$ to left of the center.

For $\alpha L \geq r_c$, we have

$$\begin{array}{rl} \tau_{\text{right}}(\alpha) = & \int_0^{r_c} dx \, x \, p(x) + \int_{r_c}^{\alpha L} dx \, (2c + \eta x) \, p(x) + \\ & \int_{\alpha L}^{R} dx \, (2c + \eta \alpha L + x - \alpha L) \, p(x) \end{array} .$$

The three integrals of the rhs of the above equation are

$$\int_0^{r_c} dx \, x \, p(x) = r_c \, G(r_c) - H(r_c) + H(0) \ ,$$

$$\begin{array}{l} \int_{r_c}^{\alpha L} dx \, (2c + \eta x) \, p(x) = 2c \, [G(\alpha L) - G(r_c)] + \\ \eta \, [\alpha L \, G(\alpha L) - r_c \, G(r_c) - H(\alpha L) + H(r_c)] \end{array}$$

and

$$\begin{array}{l} \int_{\alpha L}^{R} dx \, [2c - (1-\eta)\alpha L + x] \, g(x) = \\ {[2c - (1-\eta)\alpha L]} \, [G(R) - G(\alpha L)] + \\ R \, G(R) - \alpha L \, G(\alpha L) - H(R) + H(\alpha L) \end{array} .$$

We can thus write

$$\begin{array}{l} \tau_{\text{right}}(\alpha) = H(0) + G(r_c) \, [r_c - 2c - \eta r_c] + \\ H(r_c) \, (\eta - 1) + G(\alpha L) \, [2c + \eta \alpha L - 2c + (1-\eta)\alpha L - \alpha L] + \\ + H(\alpha L)(1-\eta) + G(R) \, [2c - (1-\eta)\alpha L + R] - H(R) \end{array}$$

from which we get

$$\begin{array}{rl} \tau_{\text{right}}(\alpha) = & H(0) + H(r_c) \, (\eta - 1) + G(R) \, (2c + R) - H(R) + \\ & (1-\eta) \, [H(\alpha L) - \alpha L \, G(R)] \, Q + \\ & (1-\eta) \, [H(\alpha L) - \alpha L \, G(R)] \end{array} , \quad \text{(S11)}$$

where

$$Q = H(0) + H(r_c) \, (\eta - 1) + G(R) \, (2c + R) - H(R) \quad \text{(S12)}$$

does not depend on $\alpha$. Please note that we use the fact that $(1-\eta) r_c - 2c = 0$ and $2c + \eta \alpha L - 2c + (1-\eta)\alpha L - \alpha L = 0$.

For $\alpha L \leq r_c$, we can instead simply write

$$\tau_{\text{right}}(\alpha) = \int_0^{R} dx \, x \, p(x) = R \, G(R) - H(R) + H(0) \ . \quad \text{(S13)}$$

Since $p(x)$ is an even function, we can write that

$$\tau_{\text{left}}(\alpha) = \tau_{\text{right}}(1-\alpha) \ . \quad \text{(S14)}$$

and

$$\tau(\alpha) = \tau_{\text{left}}(\alpha) + \tau_{\text{right}}(\alpha) \quad \text{(S15)}$$

If we take the derivative with respect to $\alpha$, we have

$$\begin{array}{rl} \frac{d}{d\alpha} \tau_{\text{right}}(\alpha) = & (1-\eta) \, L \, [H'(\alpha L) - G(R)] \\ & (1-\eta) \, L \, [G(\alpha L) - G(R)] \leq 0 \end{array} .$$

and

$$\frac{d}{d\alpha} \tau_{\text{left}}(\alpha) = -(1-\eta) L \, \{G[(1-\alpha)L] - G(R)\} \geq 0 \ .$$

The above inequalities follow from fact that $p(x)$ is a non-negative function.

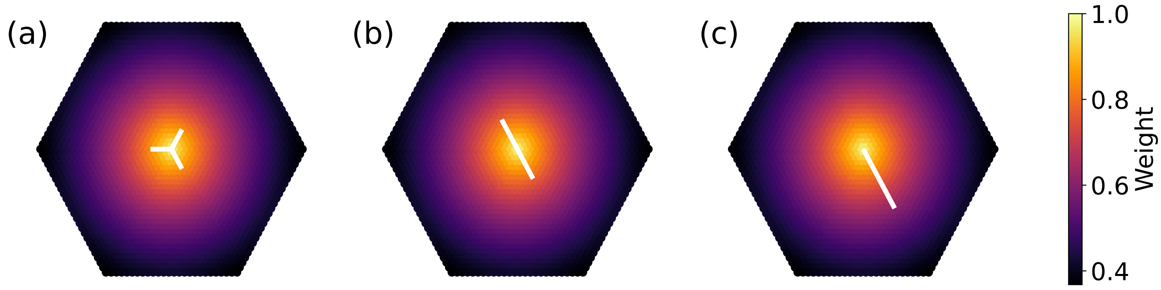


FIG. S4. **Two-dimensional triangular lattices with exponentially decaying weights.** (a) Optimal solution for the system with the fast layer composed of three branches. We use $R = 25$, $c = 0.1$, $\eta = 0.1$, and $L = 12$. The weight associated to the individual nodes in the slow layer is represented by the color map. (b) Same as in (a), but for $c = 0.7$. (c) Same as in (b), but for $c = 1.2$.

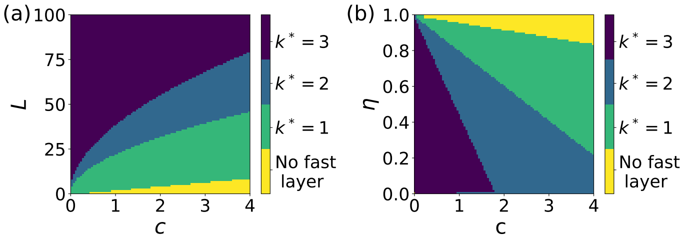


FIG. S5. **Phase diagrams for optimal fast-layer configurations in two-dimensional triangular lattices with exponentially decaying weights.** (a) We show the heat map illustrating the type of optimal solutions obtained for different combinations of $c$ and $L$. We consider a slow layer with $R = 100$ and set the weight of the edges in the fast layer to be $\eta = 0.1$. We plot $k^*$ as a function of $c$ and $L$. The yellow region indicates that it is not beneficial to build a fast layer for the values of $c$ and $L$ as it will not reduce the minimum-cost path to the center regardless of the structure, i.e., $L < r_c$. (b) same as in (a), but the y-axis is changed to $\eta$ and the size of the fast layer is set to $L = 50$.

*Analysis*

We now suppose that $r_c/L \leq 1/2$. We have

$$\tau(\alpha) = \begin{cases} \tau_{\text{right}}(0) + \tau_{\text{left}}(1-\alpha) & \text{if } 0 \leq \alpha \leq r_c/L \\ \tau_{\text{right}}(\alpha) + \tau_{\text{right}}(1-\alpha) & \text{if } r_c/L \leq \alpha \leq 1/2 \end{cases} . \tag{S16}$$

If we take the derivative with respect to $\alpha$, we get

$$\frac{d\,\tau(\alpha)}{d\alpha} = \begin{cases} -(1-\eta)L\left\{G[(1-\alpha)L] - G(R)\right\} \geq 0 & \text{if } 0 \leq \alpha \leq r_c/L \\ (1-\eta)L\left\{G(\alpha L) - G[(1-\alpha)L]\right\} \leq 0 & \text{if } r_c/L \leq \alpha \leq 1/2 \end{cases} . \tag{S17}$$

Thus $\tau(\alpha)$ reaches its maximum at $\alpha = r_c/L$. The function is instead minimized either for $\alpha = 0$ or $\alpha = 1/2$.

The same result is obtained $r_c/L \geq 1/2$. In such a case, the maximum of the function $\tau(\alpha)$ is reached for $\alpha = 1 - r_c/L$.

*Critical condition*

The transition between the solution $\alpha = 0$ and $\alpha = 1/2$ is obtained by simply solving the equation $\tau(0) = \tau(1/2)$.

We have

$$\tau(0) = \tau_{\text{right}}(0) + \tau_{\text{left}}(1)$$

and

$$\tau(1/2) = 2\tau_{\text{right}}(1/2) .$$

Imposing the critical condition, we get

$$(1-\eta)\,H(r_c) - 2c\,G(R) - 2(1-\eta)\,H(L/2) = 0 .$$

which can be rewritten as

$$H(r_c) - r_c\,G(R) - 2H(L/2) = 0 . \tag{S18}$$

*Special cases*

If $p(x) = 1$, the $G(x) = x$ and $H(x) = x^2/2$, Eq. (S18) becomes

$$r_c^2/2 - r_c R - L^2/4 = 0 \, ,$$

thus

$$r_c^2 - 2R\,r_c - L^2/2 = 0$$

from which we get

$$r_c^\dagger = R - \sqrt{R^2 - L^2/2} \, , \tag{S19}$$

that is Eq. (10) of the main manuscript.

If $p(x) = x^{-\gamma}$, the $G(x) = \frac{x^{-\gamma+1}}{-\gamma+1}$ and $H(x) = \frac{x^{-\gamma+2}}{(-\gamma+2)(-\gamma+1)}$, Eq. (S18) becomes

$$\frac{r_c^{-\gamma+2}}{(-\gamma+2)(-\gamma+1)} - r_c \frac{R^{-\gamma+1}}{-\gamma+1} - 2^{\gamma-1} \frac{L^{-\gamma+2}}{(-\gamma+2)(-\gamma+1)} = 0$$

thus

$$\frac{r_c^{-\gamma+2}}{(-\gamma+2)} - r_c\,R^{-\gamma+1} - 2^{\gamma-1} \frac{L^{-\gamma+2}}{(-\gamma+2)} = 0$$

If $p(x) = e^{-x/V}$, the $G(x) = -Ve^{-x/V}$ and $H(x) = V^2 e^{-x/V}$, Eq. (S18) becomes

$$Ve^{-r_c/V} + r_c e^{-R/V} - 2Ve^{-L/2V} = 0 \, .$$

## B. Star-like systems

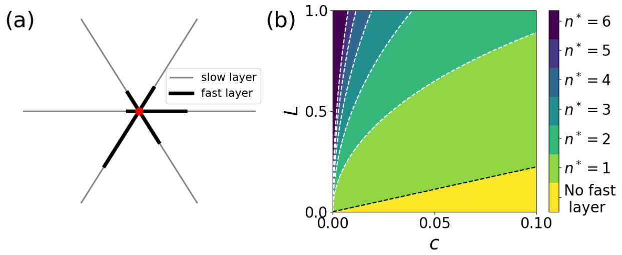


FIG. S6. **Star-like systems**. (a) Slow layer composed of $q = 6$ branches with a fast layer composed of $n = 6$ branches of unequal length. The red circle shows the center of the system. (b) Optimal solutions for the system shown in (a) for different values of $L$ and $c$ and fixed $R = 1$ and $\eta = 0.1$. The dashed white curves show the analytically obtained boundaries between different solutions, i.e., Eq. (S22), and the black dashed line shows the region where no fast layer solution exists, i.e., $L \leq r_c$.

We consider a slow layer consisting of $q$ branches of length $R$ emerging from the center $o$. The optimal fast layer must be a tree rooted in the replica of the center with $1 \leq n^* \leq q$ branches (see Figure S6). In this setting, we can show that the length of these $n^*$ branches must be equal.

Let us assume that this is not true and the optimal solution consists of branches of potentially unequal lengths. Consider two of them of length $\ell_1$ and $\ell_2$, respectively. We can map this configuration to the one-dimensional case by simply using $L = \ell_1 + \ell_2$. This automatically tells us that the configuration in which $\ell_1 = \ell_2$ is better than any configuration where $\ell_1 \neq \ell_2$. We note in fact that the inequality $r_c \geq r^\dagger$ is already satisfied, otherwise, the optimal solution would have consisted of a number of branches different from $n^*$.

The above consideration is valid for any pair selected out of the $n^*$ branches, therefore, the optimal solution in this setting should be given by branches of equal length.

We can obtain the boundaries between the optimal fast-layer solutions for this system by extending the framework used in the one-dimensional case. As shown above, the solutions can be characterized by the number of branches $n^*$ of equal length $L/n^*$. Similar to Eq.(7) of the main paper, the average cost to reach the center for a single branch with a fast layer of length $\ell$ attached to the center is given by

$$\tau_{\text{single}}(\ell) = \begin{cases} R^2/2 & \text{if } 0 \leq \ell \leq r_c \\ C + (1-\eta)\ell\,[\ell/2 - R] & \text{oth.} \end{cases} \, , \tag{S20}$$

where

$$C = \frac{1}{2}(1-\eta)r_c^2 + 2c(R - r_c) + \frac{1}{2}R^2 \, . \tag{S21}$$

Therefore, the average cost to reach the center for a generic solution with $n^*$ branches can be written using Eq. S20 as

$$\tau(L, n^*) = n^* \tau_{\text{single}}(L/n^*) + (q - n^*)\tau_{\text{single}}(0) \tag{S22}$$

Consequently, the boundary between solutions with $n^*$ and $n^* + 1$ branches is given by the condition $\tau(L, n^*) = \tau(L, n^* + 1)$. We obtain the following critical values

$$L^\dagger(n^*) = \sqrt{n^*(n^*+1)(2Rr_c - r_c^2)} \, . \tag{S23}$$

The obtained boundaries alongside the optimal solutions for $\eta = 0.1$, $R = 1$, and different values of $c$ and $L$ are shown in Figure S6 b.

## C. Two-dimensional systems

We consider a circle of radius $R$. A fast layer of linear size $L$ is present. We assume that the fast layer is composed of $n$ segments or branches, each of length $L/n$. Each segment departs from the origin. Consecutive segments are separated one from the other by an angle equal

to $2\pi/n$ (see Figure S7). Please note the above assumption is reasonable, but we do not have a mathematical proof that the optimal fast layer has such a geometry. Our goal is to compute the cost of the minimum-cost path $\tau(n)$ of all points in the slow layer to the center of the circle, and then determine the number of branches of the optimal fast layer as

$$n^* = \arg\min_n \tau(n) \ . \tag{S24}$$

No fast layer is created if $R \leq r_c$. In such a case, the objective function reads

$$\tau(n = 0) = 4\tau_{\mathrm{e}} \ . \tag{S25}$$

If $R \geq r_c$, the solution should be determined by comparing the value of the objective function for different $n$ values. These can be systematically computed as follows. For $n = 1$, we have

$$\tau(n = 1) = 2\tau_{\mathrm{e}} + 2\tau_{\mathrm{f}}(L, \pi/2) \ , \tag{S26}$$

which stands for the sum of the costs of all minimum-cost paths in two empty quarters of the circle, i.e., spanning an angle equal to $\pi/2$, and of the costs of all minimum-cost paths of two quarters containing a fast layer of length $L$ located on one of the sides of the quarter of the circle. For $n > 1$, we have

$$\tau(n) = 2n\tau_{\mathrm{f}}(L/n, \pi/n) \ , \tag{S27}$$

thus the objective function is given by the sum of $2n$ identical contributions. Each contribution refers to the cost of the minimum-cost paths associated to a section of the circle that spans an angle equal to $\pi/n$ and takes advantage of a fast layer of length $L/n$ that is located on one of the sides of the section.

The quantities appearing on the r.h.s. of Eqs (S26) and (S27) can be computed by solving specific integrals. Details are reported in the following sections. From Figure S8(a), we see that as long as $c$ increases and $\eta$ is kept constant, $n^*$ decreases. We see instead from Figure S8(b), that $n^*$ increases as $\eta$ increases for fixed $c$. The result is consistent with what already observed in the one-dimensional and on ER graphs, and the heat maps of Figure S9 confirm the fact that qualitatively similar results are valid regardless of the dimensionality of the system.

In Figure S9, we plot $n^*$, as defined in Eq. S24, as a function of $\eta$ and $c$. Only values $1 \leq n \leq 6$ is considered in the numerical test.

*Empty quarter*

This is the simplest case where the fast layer is not used. We simply have

$$\tau_{\mathrm{e}} = \int_0^R dr \int_0^{\pi/2} d\theta \ r = \frac{\pi R^2}{4} \ . \tag{S28}$$

*Section of the circle spanning an angle $\phi$ with a fast layer of length $\ell$*

The fast layer is a segment going from $(0, 0)$ to $(\ell, 0)$. A generic point $(r, \theta)$, with $0 \leq r \leq R$ and $0 \leq \theta \leq \phi$ can reach the origin either using or not the fast layer. If it does not use it, then the cost of the minimum-cost path is $q_l = r$. If it does use it, then the cost is

$$q_g = \sqrt{r_g^2 + r^2 - 2r\, r_g \cos\theta} + \eta r_g + 2c \ .$$

The first term is the distance of $(r, \theta)$ to the point $(r_g, 0)$ on the fast layer that must be reached using the slow layer. The other two terms account for the cost of the path to the center on the fast layer and the penalty associated with the change of layers. We have that $r_g = \min\{\ell, r_\times\}$, with

$$r_\times = \arg\min_z \sqrt{z^2 + r^2 - 2r\, z \cos\theta} + \eta z \ . \tag{S29}$$

If $r_g = \ell$, the minimum-cost path takes advantage of the entire length of the fast layer; if $r_g = r_\times \leq \ell$, then only part of the fast layer is used by the minimum-cost path to the center. To compute $r_\times$, we just find the value for which

$$\frac{d}{dz}\left(\sqrt{z^2 + r^2 - 2r\, z \cos\theta} + \eta z\right) = 0 \ .$$

After some calculations, we arrive to the expression

$$r_\times = r\left(\cos\theta - \frac{\eta}{\sqrt{1-\eta^2}} \sin\theta\right) \ . \tag{S30}$$

We can now insert the expression of Eq. (S30) into Eq. (S29) to determine the value of $q_g$ in $r_\times$.

After some calculations, we find

$$q_g = \begin{cases} q_{g_1} & \text{if } \ell \leq r\left(\cos\theta - \frac{\eta}{\sqrt{1-\eta^2}}\sin\theta\right) \\ q_{g_2} & \text{otherwise} \end{cases} \tag{S31}$$

with

$$q_{g_1} = \sqrt{\ell^2 + r^2 - 2r\,\ell\cos\theta} + \eta\ell + 2c \tag{S32}$$

and

$$q_{g_2} = r\left(\eta\cos\theta + \sqrt{1-\eta^2}\sin\theta\right) + 2c \ . \tag{S33}$$

To compute our quantity of interest, we need to perform the integral

$$\tau_{\mathrm{f}}(\ell, \phi) = \int_0^R dr \int_0^\phi d\theta \ \min\{r, q_g\} \ . \tag{S34}$$

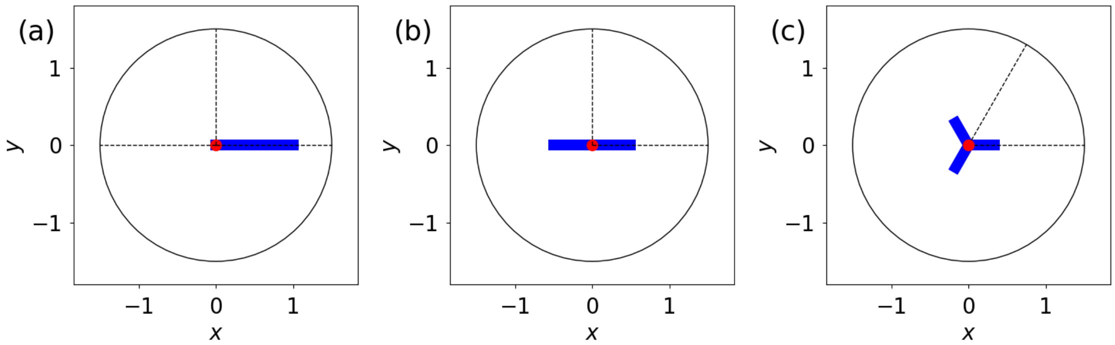


FIG. S7. (a) Circle with a fast layer composed of a single branch. Here, $R = 1.5$ and $L = 1$. The two relevant quadrants for the calculations of the objective function are delimited by the dashed lines. (b) Same as in (a), but for $n = 2$ branches. Only one quadrant is relevant for the computation of the objective function and its delimited by the dashed lines. (c) Same as in (b), but for $n = 3$ branches.

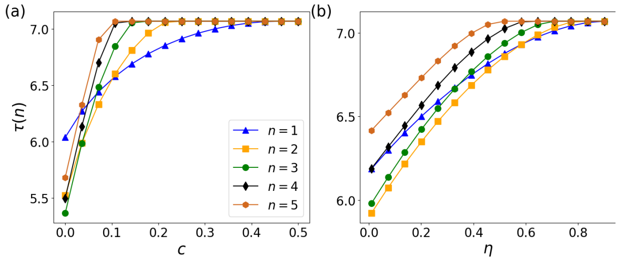


FIG. S8. (a) $R = 1.5$, $L = 1$, $\eta = 0.1$. We plot $\tau(n)$ as a function of $c$ for different $n$ values. (b) Same as in (a) but for fixed $c = 0.05$ and varying $\eta$.

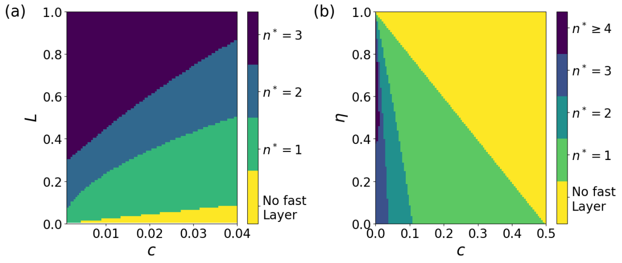


FIG. S9. (a) $R = 1.5$ and $\eta = 0.1$. For each pair of values $c$ and $\eta$, we determine the value of $n$ solution of Eq. (S24). (b) same as (a) but $L = 1$ and $c$ and $\eta$ are varied.

*Boundaries between the various regions*

In the following, we determine the equations that define the boundaries between the various regions of integration of Eq. (S34). With reference to Fig. S10, we define three distinct parts of the section of the circle: (i) the region $l$ composed of points whose minimum-cost path to the center is entirely on the slow layer; (ii) the region $g_1$ composed of points that reach the center using entire length of the fast layer; (iii) the region $g_2$ composed of points that reach the center using part of the fast layer.

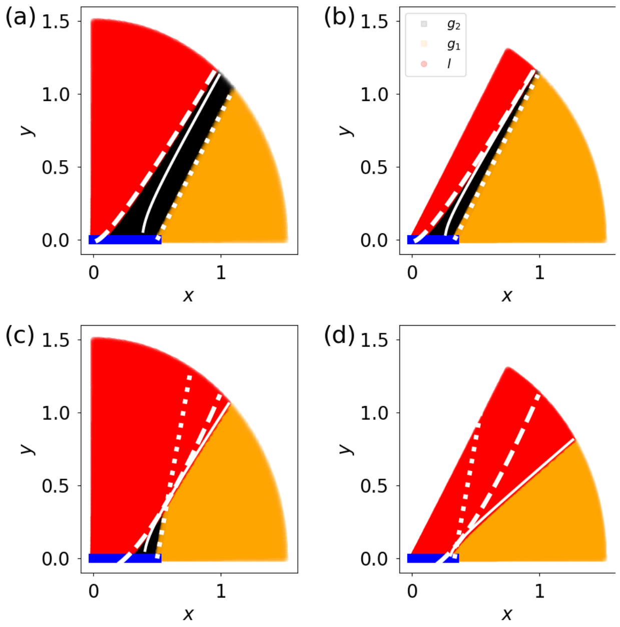


FIG. S10. (a) $R = 1.5$, $L = 1$, $\eta = 0.5$ and $c = 0.01$. We consider $n = 2$ branches, and show only the region of the circle with angular coordinate $\theta \in [0, \pi/2]$. The red area denoted as $l$ is the region of points reaching the origin without the use of the fast layer. The black region, labeled as $g_2$, corresponds to points that use the fast layer from $0 < r_\times < L/n$. The white dashed curve is given by Eq. S36. Finally, the orange region, namely $g_1$, indicates the part of the system that use the entire length of the fast layer. The dotted white curve is given by Eq. S38. The full white region is given by Eq. S40. (b) Same as in (a) but for $n = 3$. (c) Same as in (a) but for $\eta = 0.2$ and $c = 0.1$. (d) Same as in (c) but for $n = 3$.

The distinction between the $l$ and $g_2$ regions is determined by the condition $q_l = q_{g_2}$, thus

$$\eta \cos\theta_{l,g_2} + \sqrt{1-\eta^2}\sin\theta_{l,g_2} = \frac{r-2c}{r} \; . \qquad \text{(S35)}$$

After some calculations, we get

$$\theta_{l,g_2} = \arcsin \frac{r - 2c}{r} - \arcsin \eta \; . \qquad \text{(S36)}$$

The distinction between the $g_1$ and $g_2$ regions is determined by the condition $r_\times = \ell$, thus

$$-\cos \theta_{g_1,g_2} + \frac{\eta}{\sqrt{1-\eta^2}} \sin \theta_{g_1,g_2} = -\ell/r \; , \qquad \text{(S37)}$$

from which

$$\theta_{g_1,g_2} = -\arcsin \frac{\ell \sqrt{1-\eta^2}}{r} + \arcsin \sqrt{1-\eta^2} \; . \qquad \text{(S38)}$$

The distinction between the $l$ and $g_1$ regions is determined by the condition $r = q_{g_1}$, thus

$$r = \sqrt{\ell^2 + r^2 - 2r\ell \cos \theta_{l,g_2}} + \eta\ell + 2c \; . \qquad \text{(S39)}$$

After some calculations, we get

$$\theta_{l,g_1} = \frac{\pi}{2} - \arcsin \frac{1}{\ell} \left( \frac{\ell^2 - (\eta\ell + 2c)^2}{2r} + \eta\ell + 2c \right) \; . \qquad \text{(S40)}$$

## VII. REAL CITIES

In the main paper, we show the phase transition between different types of solutions in Toronto. In Figures S11 and S12, we display results of a similar analysis for Boston and Atlanta.

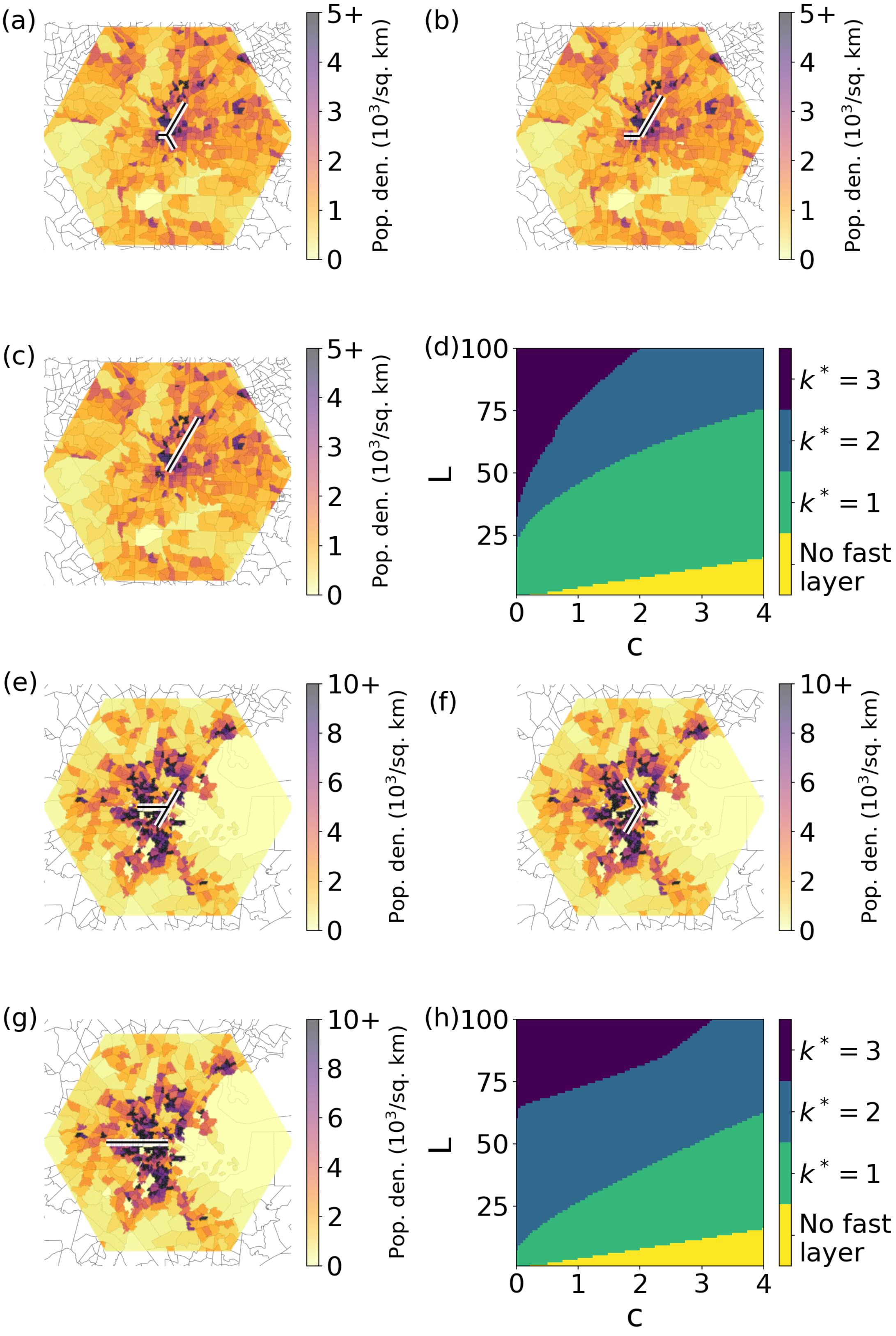


FIG. S11. **Phase transition in the optimal fast layer for Atlanta and Boston.** We set the city radius $\tilde{R} = 20$ km for Boston and $\tilde{R} = 25$ km for Atlanta, and we use a triangular lattice with radius $R = 100$ to model the layers of the multiplex transportation system. The color map displays the population density in the city. We show the distinct solutions obtained for the length of the fast layer $L = 50$ for various values of the switching cost $c$ and $\eta = 0.5$. (a) Optimal fast network with the degree of center $k^* = 1$ for Atlanta. (b) Same as in (a) with $k^* = 2$. (c) Same as in (a) with $k^* = 1$. (d) We show the heat map illustrating the type of optimal solution (shown in panels (a) and (b)) obtained for different combinations of $c$ and $L$. The yellow region indicates that building a fast layer for the values of $c$ and $L$ does affect the minimum-cost paths to the center regardless of the structure, i.e., $L < r_c$. (e) Optimal fast network with the degree of center $k^* = 1$ for Boston. (f) Same as in (e) with $k^* = 2$. (g) Same as in (e) with $k^* = 1$. (h) Same as in (d) for Boston.

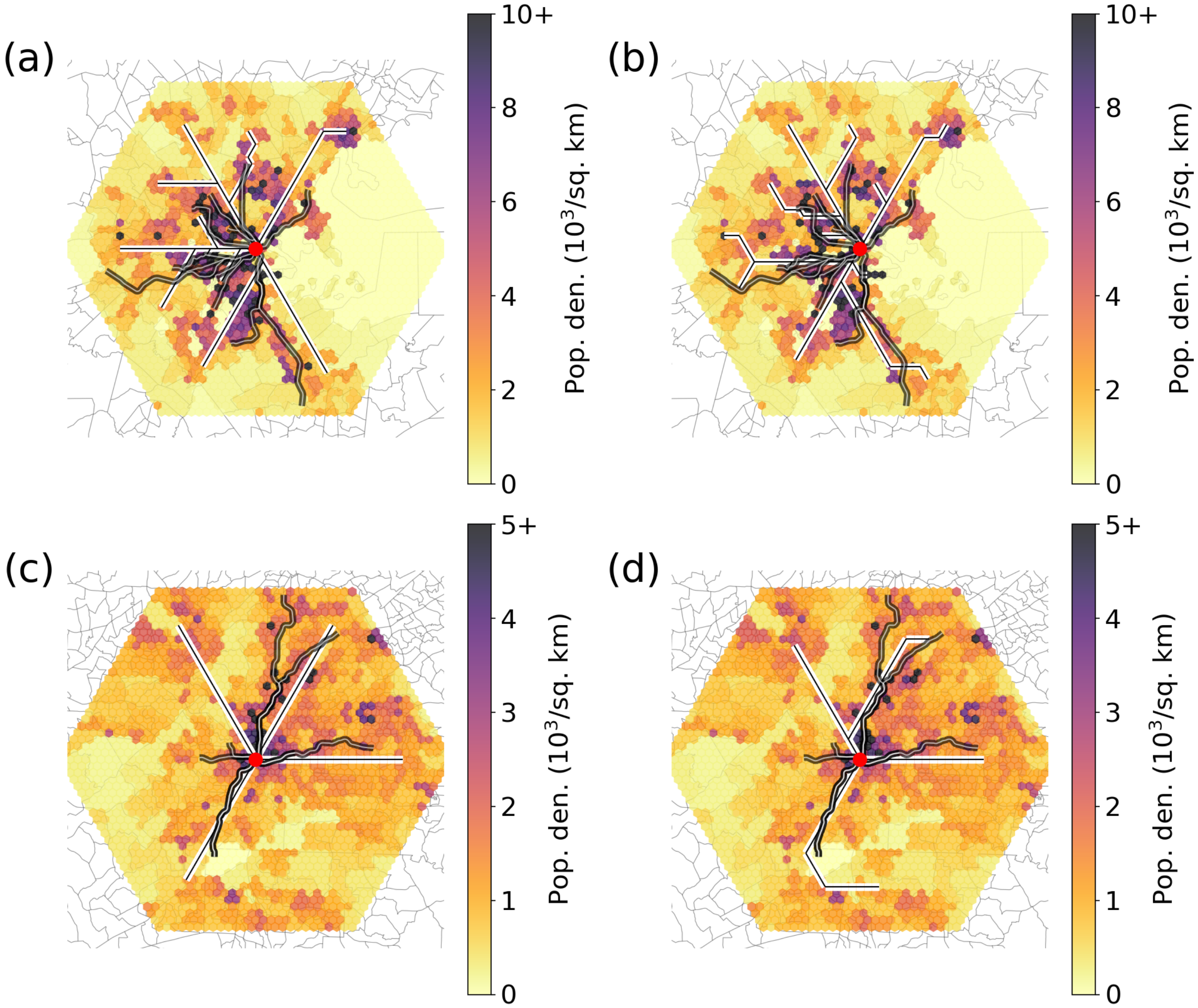


FIG. S12. **Illustration of the optimal fast layer for Atlanta and Boston.** We compare the solution obtained from our optimization algorithm (thick white lines) with the real subway system (thick black curves) in Boston (a, b) Atlanta (c, d) for two different parameter settings corresponding to low (a, c) and high (b, d) congestion. (a) The optimized configuration for Boston is obtained by setting $\eta = 0.125$ and $c = 1.25$, corresponding to a switching time of 3 minutes and slow and fast layer speeds 20 km/h and 40 km/h, respectively. (b) same as (a) but for $\eta = 0.5$ and $c = 0.3125$, corresponding to a switching time of 3 minutes and slow and fast layer speeds 5 km/h and 40 km/h, respectively. (c) The optimized configuration for Atlanta for low congestion is obtained by setting $\eta = 0.125$ and $c = 1$, corresponding to a switching time of 3 minutes and slow and fast layer speeds 20 km/h and 40 km/h, respectively. (d) same as (c) but for $\eta = 0.125$ and $c = 0.25$, corresponding to a switching time of 3 minutes and slow and fast layer speeds 5 km/h and 40 km/h, respectively. The heat maps are used to represent population densities in the cities.
