# Supplementary material for: Symmetry breaking in optimal transport networks
Source: Nat Commun. 2024 May 4;15:3758. doi: 10.1038/s41467-024-48068-9 (PMC11069546; doi:10.1038/s41467-024-48068-9)
Supplement: Supplementary file 3 — Reporting Summary [file 41467_2024_48068_MOESM3_ESM.pdf]

nature portfolio

Corresponding author(s): Filippo Radicchi

Last updated by author(s): Mar 28, 2024

Reporting Summary

Nature Portfolio wishes to improve the reproducibility of the work that we publish. This form provides structure for consistency and transparency in reporting. For further information on Nature Portfolio policies, see our Editorial Policies and the Editorial Policy Checklist.

Statistics

For all statistical analyses, confirm that the following items are present in the figure legend, table legend, main text, or Methods section.

n/a	Confirmed	
☒	☐	The exact sample size (*n*) for each experimental group/condition, given as a discrete number and unit of measurement
☒	☐	A statement on whether measurements were taken from distinct samples or whether the same sample was measured repeatedly
☒	☐	The statistical test(s) used AND whether they are one- or two-sided<br>*Only common tests should be described solely by name; describe more complex techniques in the Methods section.*
☒	☐	A description of all covariates tested
☒	☐	A description of any assumptions or corrections, such as tests of normality and adjustment for multiple comparisons
☒	☐	A full description of the statistical parameters including central tendency (e.g. means) or other basic estimates (e.g. regression coefficient) AND variation (e.g. standard deviation) or associated estimates of uncertainty (e.g. confidence intervals)
☒	☐	For null hypothesis testing, the test statistic (e.g. *F*, *t*, *r*) with confidence intervals, effect sizes, degrees of freedom and *P* value noted<br>*Give P values as exact values whenever suitable.*
☒	☐	For Bayesian analysis, information on the choice of priors and Markov chain Monte Carlo settings
☒	☐	For hierarchical and complex designs, identification of the appropriate level for tests and full reporting of outcomes
☒	☐	Estimates of effect sizes (e.g. Cohen's *d*, Pearson's *r*), indicating how they were calculated

Our web collection on statistics for biologists contains articles on many of the points above.

Software and code

Policy information about availability of computer code

Data collection	We obtain the population density at the census-tract level for Boston and Atlanta from the 2021 American Census Survey, and for Toronto from the 2021 Canadian Census of Population. The census data contains the number of individuals residing in relatively small geographic regions, i.e., census tracts, used for statistical purposes by national statistical agencies. Census tracts typically consist of $2,500$ to $8,000$ individuals.<br><br>The data on the four metro lines and their stations in Boston is obtained from the Massachusetts Bureau of Geographic Information (MassGIS) website. Similar data for the four metro lines in Atlanta is obtained from the Atlanta Regional Commission: Open Data website.<br>Finally, the data for the three subway lines and their stations in Toronto is made available by the Toronto Transit Commission at the City of Toronto Open Data website.<br><br>The data on the subway lines is available as shapefiles. The arcs for the rail lines are given by sets of points in the coordinate reference systems (CRS) applicable to the geographic location. The CRSs used for Atlanta, Boston, and Toronto are EPSG:2239, EPSG:26986, and, EPSG:2952, respectively.
Data analysis	No special software was used to analyze the data

For manuscripts utilizing custom algorithms or software that are central to the research but not yet described in published literature, software must be made available to editors and reviewers. We strongly encourage code deposition in a community repository (e.g. GitHub). See the Nature Portfolio guidelines for submitting code & software for further information.

Data

Policy information about availability of data

All manuscripts must include a data availability statement. This statement should provide the following information, where applicable:

- Accession codes, unique identifiers, or web links for publicly available datasets
- A description of any restrictions on data availability
- For clinical datasets or third party data, please ensure that the statement adheres to our policy

The datasets analyzed during the current study are available at: Census Reporter, https://censusreporter.org/, Toronto Transit Commission, https://open.toronto.ca/dataset/ttc-subway-shapefiles/, Atlanta Regional Commission, https://opendata.atlantaregional.com/search?collection=Dataset, and MassGIS, Boston rail data, https://www.mass.gov/info-details/massgis-data-trains

Research involving human participants, their data, or biological material

Policy information about studies with human participants or human data. See also policy information about sex, gender (identity/presentation), and sexual orientation and race, ethnicity and racism.

Reporting on sex and gender	N/A
Reporting on race, ethnicity, or other socially relevant groupings	N/A
Population characteristics	N/A
Recruitment	N/A
Ethics oversight	N/A

Note that full information on the approval of the study protocol must also be provided in the manuscript.

Field-specific reporting

Please select the one below that is the best fit for your research. If you are not sure, read the appropriate sections before making your selection.

☐ Life sciences ☒ Behavioural & social sciences ☐ Ecological, evolutionary & environmental sciences

For a reference copy of the document with all sections, see nature.com/documents/nr-reporting-summary-flat.pdf

Behavioural & social sciences study design

All studies must disclose on these points even when the disclosure is negative.

Study description	We used publicly available data regarding population density of two cities in the US and one city in Canada.
Research sample	N/A
Sampling strategy	N/A
Data collection	Data are publicly available. Sources are cited in the data availability statement.
Timing	N/A
Data exclusions	N/A
Non-participation	N/A
Randomization	N/A

Reporting for specific materials, systems and methods

We require information from authors about some types of materials, experimental systems and methods used in many studies. Here, indicate whether each material, system or method listed is relevant to your study. If you are not sure if a list item applies to your research, read the appropriate section before selecting a response.

Materials & experimental systems

n/a	Involved in the study
☒	☐ Antibodies
☒	☐ Eukaryotic cell lines
☒	☐ Palaeontology and archaeology
☒	☐ Animals and other organisms
☒	☐ Clinical data
☒	☐ Dual use research of concern
☒	☐ Plants

Methods

n/a	Involved in the study
☒	☐ ChIP-seq
☒	☐ Flow cytometry
☒	☐ MRI-based neuroimaging

Plants

Seed stocks	N/A
Novel plant genotypes	N/A
Authentication	N/A
